# UHRF1 suppression promotes cell differentiation and reduces inflammatory reaction in anaplastic thyroid cancer

**DOI:** 10.18632/oncotarget.10674

**Published:** 2016-07-18

**Authors:** Bi-Cheng Wang, Guo-He Lin, Bo Wang, Min Yan, Bin He, Wei Zhang, An-Kui Yang, Zi-Jie Long, Quentin Liu

**Affiliations:** ^1^ State Key Laboratory of Oncology in South China, Collaborative Innovation Center for Cancer Medicine, Cancer Center, Sun Yat-sen University, Guangzhou, 510060, China; ^2^ Department of Medical Oncology, The Eastern Hospital of The First Affiliated Hospital, Sun Yat-sen University, Guangzhou, 510700, China; ^3^ Department of Hematology, The Third Affiliated Hospital, Sun Yat-sen University, Guangzhou, 510630, China; ^4^ Institute of Hematology, Sun Yat-sen University, Guangzhou, 510630, China; ^5^ Institute of Cancer Stem Cell, Dalian Medical University, Dalian, 116044, China

**Keywords:** UHRF1, anaplastic thyroid cancer, proliferation, differentiation, inflammatory reaction

## Abstract

Anaplastic thyroid cancer (ATC), an undifferentiated subtype of thyroid cancer, is one of the most malignant endocrine cancer with low survival rate, and resistant to chemotherapy and radiation therapy. Here we found that UHRF1 was highly expressed in human ATC compared with normal tissue and papillary thyroid cancer (PTC). Knockdown of UHRF1 inhibited proliferation of ATC *in vitro* and *in vivo*. Consistently, overexpression of UHRF1 promoted the proliferation of thyroid cancer cells. Moreover, UHRF1 suppression induced differentiation of three-dimensional (3D) cultured ATC cells and down-regulated the expression of dedifferentiation marker (CD97). The stem cell markers (Sox2, Oct4 and Nanog) were suppressed simultaneously. In addition, UHRF1 knockdown reduced the transcription of cytokines (IL-8, TGF-α and TNF-α), which might relieve the inflammatory reaction in ATC patients. This study demonstrated a role of UHRF1 in ATC proliferation, dedifferentiation and inflammatory reaction, presenting UHRF1 as a potential target in ATC therapy.

## INTRODUCTION

Anaplastic thyroid cancer (ATC) has a poor prognosis with only half a year survival lifespan after diagnosis [1, 2]. Regional lymph nodal invasion and distant metastasis have been found in over 60% patients [2, 3]. ATC exhibits faster growth properties in comparison with well-differentiated thyroid cancer. Currently, surgery and chemo-/radio-therapies are not effective in ATC [3–6].

ATC is also known as an undifferentiated thyroid cancer. During the dedifferentiation process, ATC loses the normal thyroid ability, including the ability to express the thyroid stimulating hormone (TSH) receptor, leading to the resistance to TSH suppressive treatment. Differentiation therapy in ATC aims to regain the thyroid-specific structure and function. Recently, ATRA and histone deacetylase inhibitors (HDAC inhibitors) provide a differentiation therapy option for ATC. However, the effectiveness of these drugs for ATC patients is limited [7, 8]. Thus, additional therapies are needed and identifying novel molecular targets underlying ATC tumorigenesis is crucial for the development of ATC therapy.

UHRF1, also known as Np95 and ICBP90, a ubiquitin-like with PHD and ring-finger domain 1 protein, has four important domains, including NIRF_N domain (ubiquitin-like domain), the Plant Homeodomain domain (PHD domain), Set and Ring Associated domain (SRA domain) and Really Interesting New Gene finger domain (RING domain). UHRF1 is expressed at a constant high level in cancer cells [9, 10], including breast cancer [11, 12], lung cancer [13, 14], bladder cancer [15], laryngeal squamous cell carcinoma [16], hepatocellular carcinoma [17], and esophageal squamous cell carcinoma [18]. The overexpressed UHRF1 in cancer cells led to an increased expression of oncogene (topoisomerase IIα) and decreased expression of tumor suppressor genes (RB1, p16INK4A and p14ARF) [19]. which gave rise to the proliferation capability [20, 21]. Conversely, cells depleted UHRF1 easily suffered from ionizing radiation and UV light induced DNA damage [22, 23]. Thereby, knockdown of UHRF1 may contribute to the treatment for ATC. However, the potential function of UHRF1 in the ATC differentiation therapy has not yet been elucidated.

Inflammatory reaction is a complicated and auto-regulated process. In the tumor microenvironment, large amounts of cytokines and growth factors are secreted by cancer cells or infiltrated immune cells. It has been found that undifferentiated thyroid cancer originated cell lines secreted high levels of IL-6, TGF-α and TNF-α [24, 25]. Moreover, both IL-6 and IL-8 were demonstrated as proinflammatory mediators [26]. In the inflammatory process, the risk of oncogene activation and tumor suppressor gene inactivation is increased, which may lead to the transformation of ATC [27, 28]. Hence, inhibition of the inflammation may contribute to the ATC therapy.

In the present study, we aimed to investigate the contributions of UHRF1 suppression in ATC proliferation, differentiation and inflammatory reaction.

## RESULTS

### UHRF1 is highly expressed in thyroid cancer, especially the ATC

**Figure 1:**
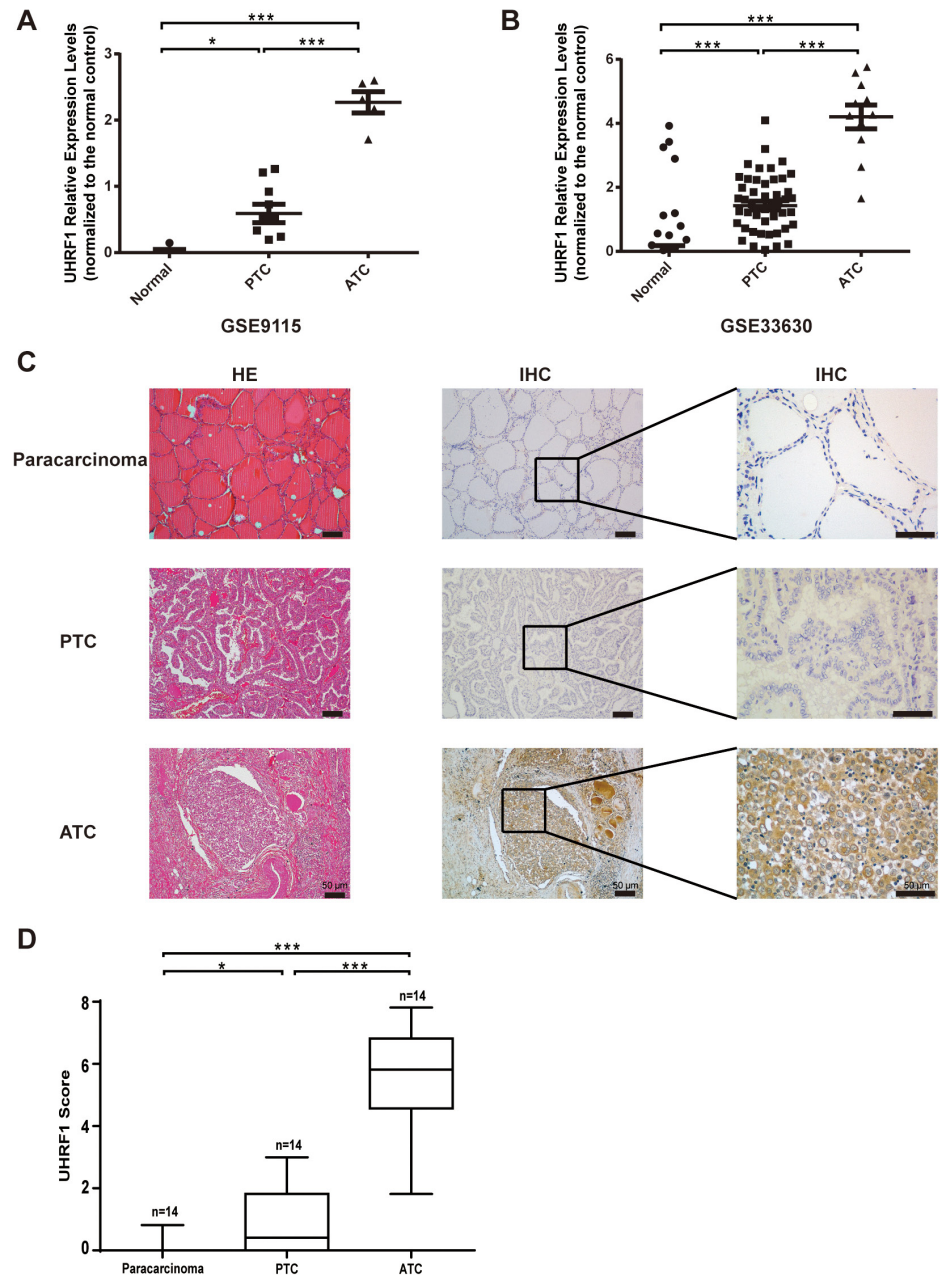
UHRF1 was highly expressed in ATC, compared with paracarcinoma and PTC **A.** and **B.** Relative expression levels of UHRF1 in microarray data GSE9115 (A) and GSE33630 (B), which were normalized to the respective normal controls. *p<0.05, ***p<0.001, two-tailed Student's t-test. **C.** Paracarcinoma tissue, PTC samples and ATC samples were collected and subjected to HE staining and IHC assay with an UHRF1 antibody. Scale bar, 50 μm. **D.** Expression level of URHF1 was compared among paracarcinoma tissue, PTC and ATC samples. Scores were determined as described in the MATERIALS AND METHODS and normalized to the control. *p<0.05, ***p<0.001, two-tailed Student's t-test.

In order to study the role of UHRF1 in thyroid cancer, especially the ATC, we searched the public data in NCBI (GEO) and selected two microarray chips, GSE9115 [29], which contained 4 normal thyroid tissues, 9 PTC and 5 ATC samples, and GSE33630 [30, 31], which contained 45 normal thyroid tissues, 49 PTC and 11 ATC samples. Through analyzing with GEO2R on the website, we found that samples collected from thyroid cancer patients displayed a significantly increased UHRF1 level compared with normal controls. Furthermore, the expression level in ATC was much higher than in PTC (Figure 1A and 1B). To further confirm the results of public microarray data, 14 paracarcinoma tissues, 14 PTC and 14 ATC samples were conducted to hematoxylin-eosin (HE) staining and immunohistochemistry (IHC) assay (Figure 1C). We found that ATC did not show the regular structure of normal thyroid follicle and the nest cancer cells exhibited strong staining for UHRF1, compared with paracarcinoma tissue and PTC. The UHRF1 score statistics results were normalized and shown in Figure 1D. Supplementary Table S1 indicated a significant positive correlation between UHRF1 expression and differentiation status. Taken together, these data implied that UHRF1 might be a potential diagnosis marker of ATC.

### Suppression of UHRF1 inhibits ATC cell proliferation

**Figure 2:**
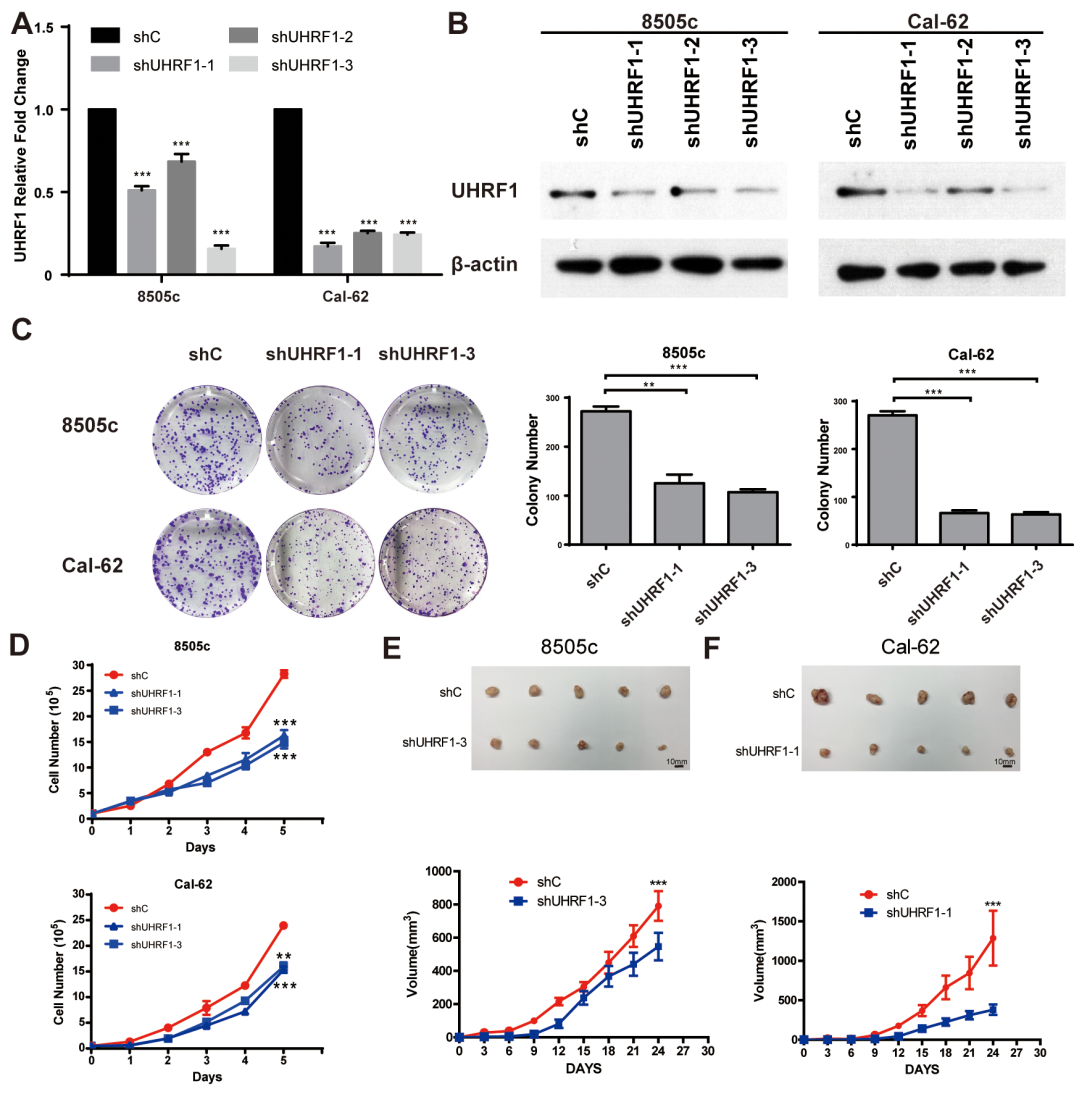
Suppression of UHRF1 inhibits the proliferation ability of thyroid cancer cells *in vitro* and *in vivo* **A.** ATC cell lines, 8505c and Cal-62, were stably transfected with shRNAs or a vector control. Real-time RT-PCR was used to validate UHRF1 expression. (***p<0.001 by two-tailed Student's t test). **B.** Western blot was used to validate the protein expression of UHRF1. **C.** The proliferation capacity of constructed stable cell lines 8505c and Cal-62 were analyzed by colony formation assay. Representative pictures of the whole plates from triplicate experiments are shown. Error bars indicate mean ± SD (**p<0.01, ***p< 0.001 by two-tailed Student's test). **D.** Approximately 1×10^5^ cells were seeded in each well of six-well plates. Cells were collected and counted by hemocytometer every 24 hours. All experiments were performed in triplicate. Error bars indicate mean ± SD, ***p< 0.001 by two-tailed Student's t test. **E.** and **F.** Nude mice were injected with 8505c shC, 8505c shUHRF1-3 (E), Cal-62 shC and Cal-62 shUHRF1-1 (F). Tumors were removed after 24 days. The average sizes of xenograft tumors measured every 3 days (n=5, error bars indicate mean ± SD, ***p< 0.001 by two-tailed Student's t test). Scale bar, 10 mm.

To examine the possible role of UHRF1 in thyroid cancer progression, we stably knocked down the expression of UHRF1 in ATC cell lines, 8505c and Cal-62. We verified the suppression effects of UHRF1 in cancer cells by real-time RT-PCR and western blot assay (Figure 2A and 2B). Strikingly, in two cancer cells tested, suppression of UHRF1 successfully inhibited the cell proliferation ability as judged by colony formation (Figure 2C). Moreover, down-regulated expression of UHRF1 dramatically suppressed the growth of ATC cell lines by cell viability assay (Figure 2D). Furthermore, we determined whether UHRF1 suppression could inhibit tumor growth in a xenograft mouse model. Consistent with the *in vitro* results, 8505c and Cal-62 cells knocked-down with UHRF1 developed smaller tumors compared with control cells (Figure 2E and 2F). In addition, overexpression of UHRF1 in PTC cell line, BCPAP, enhanced the cell proliferation (Supplementary Figure S1). Together, these findings indicated that UHRF1 was critical to the proliferation of human ATC cells and that UHRF1 inhibition reduced thyroid cancer progression.

### Knockdown of UHRF1 changes the 3D structure and reduces stemness of ATC cells

**Figure 3:**
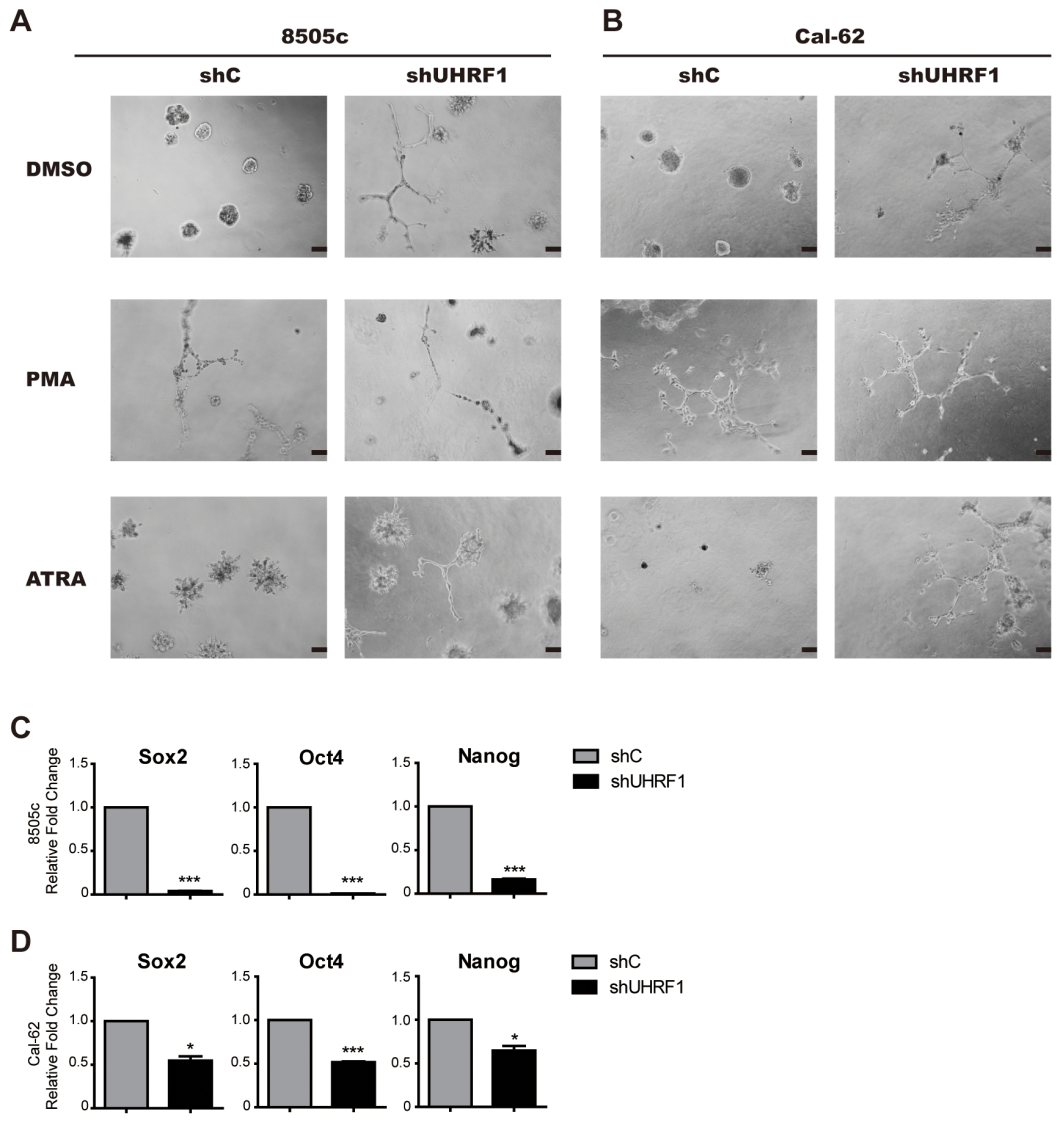
Down-regulated UHRF1 changes the structure in 3D culture and reduced the stemness of ATC cells **A.** 3D structure of 8505c shC and 8505c shUHRF1 cultured in medium containing PMA (10 ng/ml) or ATRA (1μmol). Scale bar, 100 μm. **B.** 3D structure of Cal-62 shC and Cal-62 shUHRF1 cultured in medium containing PMA (10 ng/ml) or ATRA (1μM). Scale bar, 100 μm. **C.** and **D.** Validation of stem cell markers (Sox2, Oct4 and Nanog) expression in 8505c (C) or Cal-62 (D) cell lines by real-time RT-PCR. The mean ± SD of relative fold changes from triplicate experiments was plotted. *p<0.05, ***p< 0.001 by two-tailed Student's t test.

To detect whether the expression of UHRF1 was correlated with the differentiation of thyroid cancer, UHRF1 stably knocked down thyroid cancer cell lines and control cell lines were used to perform the 3D culture assay. As shown in Figure 3A and 3B, spheroid cell clusters were formed in the control group, while the follicular-like structure was seen in the groups when UHRF1 was down-regulated. The control cells cultured with 10 ng/ml PMA [32] or 1 μM ATRA [33] were used as positive control. In the PMA group, the same follicular-like structure was exhibited. Although the similar follicular-like structure in ATRA treated control groups were not obvious, cells presented defected function of forming spheroid cell clusters. Moreover, after UHRF1 was knocked down, cells in the ATRA treated groups exhibited follicular-like structure. This data suggested that UHRF1 inhibition might induce the differentiation of undifferentiated thyroid cancer.

We next examined whether the suppression of UHRF1 could down-regulate the stemness of cancer cells. We found that stem cell markers, Sox2, Oct4 and Nanog, were strikingly decreased after knocking down of UHRF1 both in mRNA level (Figure 3C and 3D) and protein level (Supplementary Figure S2). This result was consistent with the observation in 3D culture, indicating that reduced UHRF1 expression could prohibit human thyroid cancer progression and was associated with cell differentiation.

### UHRF1 suppression decreased the expression of dedifferentiation marker CD97 after PMA or ATRA treatment

**Figure 4:**
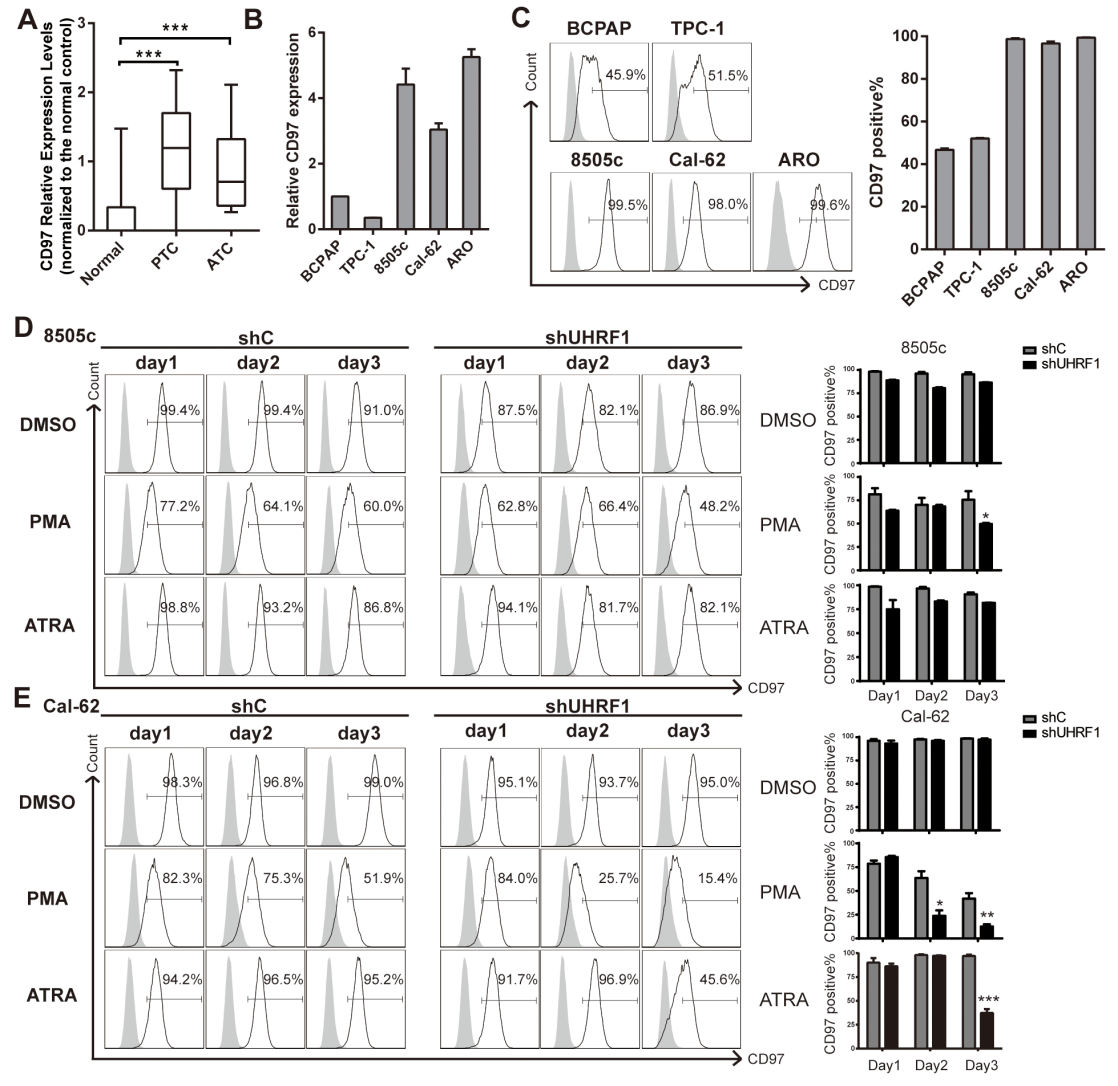
UHRF1 suppression combined with PMA or ATRA treatment effectively down-regulates the dedifferentiation marker CD97 **A.** CD97 Relative Expression Levels in microarray data GSE33630, which were normalized to the normal controls. (***p<0.001, values were calculated by two-tailed Student's test). **B.** mRNA expression of PTC cell lines (BCPAP and TPC-1) and ATC cell lines (8505c, Cal-62 and ARO) were assayed by real-time RT-PCR. **C.** Five thyroid cancer cell lines were collected for flow cytometric analysis to measure the CD97 positive population. The right panel shows the statistical results of the CD97 positive percentage measurement. The data are presented as mean ± SD of three independent experiments. **D.** and **E.** Stable cell lines, 8505c shC, 8505c shUHRF1-3(D), Cal-62 shC and Cal-62 shUHRF1-1 (E) were treated with DMSO, PMA (10 ng/ml) or ATRA (1μM) for the indicated time. Adherent cells were then subjected to flow cytometric analysis to measure the CD97 population. The right panel shows the statistical results of the CD97 positive percentage measurement. Error bars represent mean ± SD of three independent experiments (*p<0.05, **p<0.01, ***p<0.001 by two-tailed student's t test).

According to the study of Gabriela et al [32], CD97, a novel subfamily of seven-span transmembrane region leukocyte cell surface molecules, can be served as a dedifferentiation marker of human thyroid cancers. CD97 expression was tested in GSE33630 dataset, higher CD97 expression was observed in PTC and ATC tissues than normal controls (Figure 4A). Then we examined CD97 transcription in 3 paired paracarcinoma and PTC tissues. CD97 expression was about two folds higher in PTC than that in controls (Supplementary Figure S3). Moreover, we detected the CD97 levels in thyroid cancer cell lines, including two PTC cell lines (BCPAP and TPC-1) and three ATC cell lines (8505c, Cal-62 and ARO). The expression of CD97, normalized by BCPAP, is significantly higher in ATC than PTC cell lines (Figure 4B). Consistently, by flow cytometric assay, we found that the percentage of the cell population with positive CD97 expression was indeed much higher in ATC cell lines than PTC cell lines (Figure 4C). These results coincided with the studies of other group that CD97 could be used as a dedifferentiation marker [34]. Next, ATC cells were cultured with 10 ng/ml PMA or 1 μM ATRA as the indicated time and subjected to flow cytometric assay. CD97 was critically inhibited after 3 days treatment of PMA or ATRA in ATC cells when UHRF1 was suppressed (Figure 4D and 4E). After cells were treated with PMA for 3 days, we detected a lower proportion of CD97 positive cells in 8505c shUHRF1 group (49.63 ± 1.102 *vs.* shC values of 75.57 ± 9.125%, *p*<0.05) and in Cal-62 shUHRF1 group (12.57 ± 2.305 *vs.* shC values of 41.90 ± 5.717%, *p*<0.01). The discordance in ATRA treated groups might be due to the BRAF mutation in 8505c but not Cal-62 [35]. Together, these results indicated that UHRF1 suppression could promote the differentiation of ATC.

### Reduced UHRF1 expression suppressed the inflammatory reaction through down-regulating the cytokines in ATC

**Figure 5:**
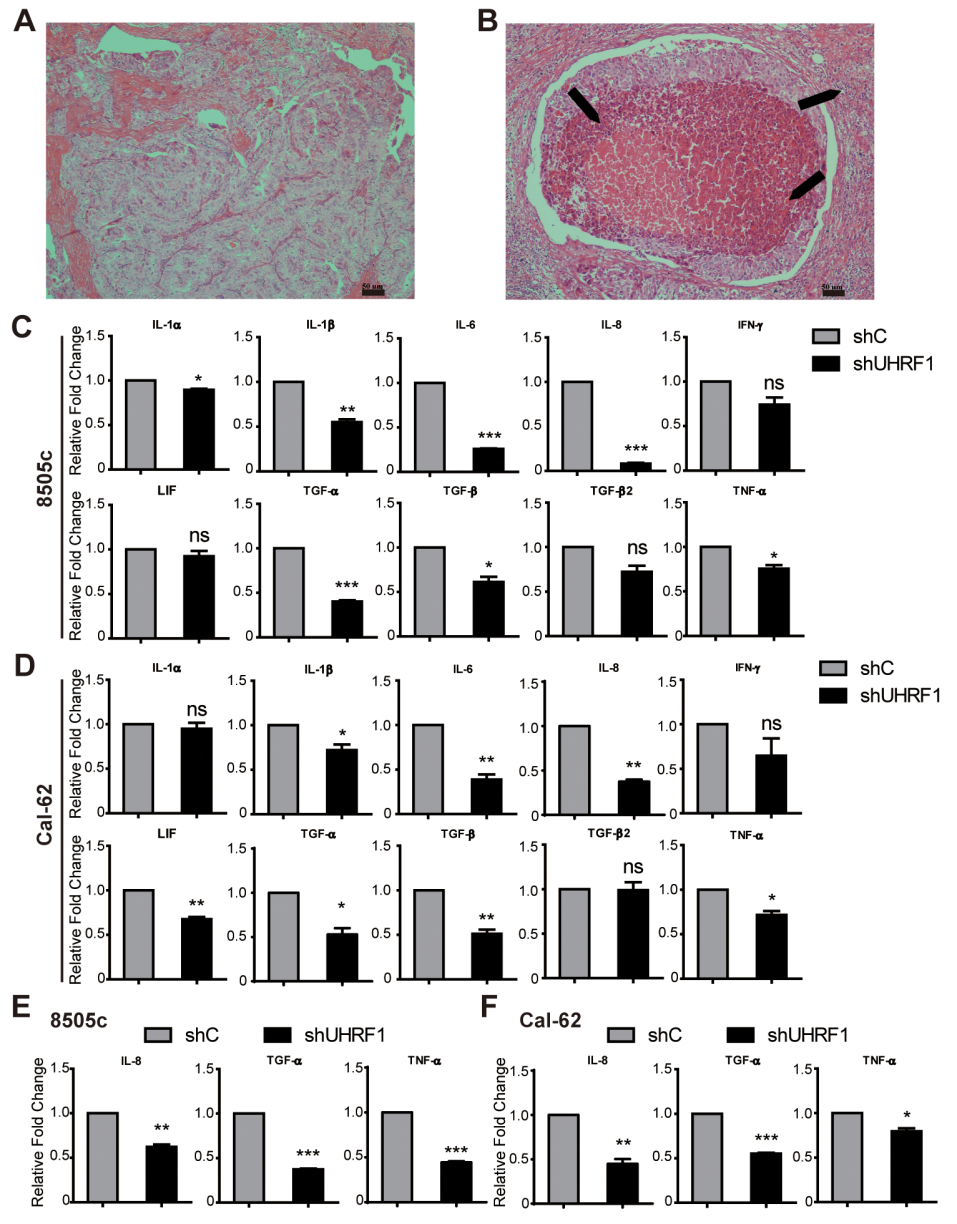
Down-regulated UHRF1 suppresses the inflammatory reaction of ATC **A.** and **B.** HE staining of PTC (A) and ATC (B). Arrows are indicated the immersed lymphocytes. Scale bar, 50 μm. **C.** and **D.** Validation of gene expression between shC and shUHRF1 in 8505c (C) or Cal-62 (D) cell lines by real-time RT-PCR. (*p<0.05, **p<0.01, ***p<0.001 by two-tailed student's t test). **E.** and **F.** Validation of IL-8, TGF-α and TNF-α expression between shC group and shUHRF1 group in 8505c (E) or Cal-62 (F) xenograft tumor tissue by real-time RT-PCR. (*p<0.05, **p<0.01, ***p<0.001 by two-tailed student's t test).

In HE staining of human thyroid cancer tissues, we also found that lymphocytes immersed in ATC tissue (Figure 5B) were more than in PTC (Figure 5A). To gain further insights into regulation function of URHF1 in tumor inflammatory, real-time RT-PCR was used to explore the related cytokines. Ten cytokines were selected [36], which played a critical role in the inflammatory reaction of ATC. We found that IL-1β, IL-6, IL-8, TGF-α, TGF-β and TNF-α were significantly decreased in both UHRF1 suppressed cells (Figure 5C and 5D). Furthermore, we confirmed our data in xenograft tumor tissue, and the results showed that IL-8, TGF-α and TNF-α were attenuated in UHRF1 suppressed group (Figure 5E and 5F). Other 7 cytokines were showed no significant differences (Supplementary Figure S4A and S4B). Thus, inhibition of UHRF1 contribute to suppression of cytokines transcription (IL-8, TGF-α and TNF-α), which might relieve the inflammatory reaction in advanced thyroid cancer patients.

## DISCUSSION

Although the overall mortality rate of well-differentiated thyroid cancers (PTC and follicular thyroid cancer, FTC) remains low, ATC patients died within 6 months of diagnosis, which accounts for more than 50% of all thyroid cancer related deaths. Attempts to identify specific molecules that could allow targeted therapy are necessary. UHRF1 has been recognized recently as a major molecular marker of human cancers [17, 37], of which the overexpression is related to the poor prognosis [16]. However, whether UHRF1 is a potential therapeutic target in ATC is unclear. Therefore, an insight study of UHRF1 dysregulation in ATC should be needed.

In evaluating the expression level of UHRF1 among normal thyroid tissue, PTC and ATC, we found that UHRF1 is overexpressed in ATC other than paracarcinoma or PTC. ATC and many other cancers, of which UHRF1 were highly expressed, have the strong ability of proliferation, invasion and metastasis. In copy-number analysis, UHRF1 overexpression showed no correlation with copy-number variation at the UHRF1 locus in hepatocellular carcinoma [17]. It is suggested that a different mechanism drives UHRF1 overexpression. Several studies have shown that microRNAs (miR-146a/b and miR-9) were down-regulated in cancers with high proliferation and invasion capability. Furthermore, the UHRF1 levels in these cancers were inversely correlated with the expression of microRNAs, and restoration of the microRNAs decreased the expression of UHRF1 by targeting of its 3’-UTR directly [38, 39]. As ATC is defined as a cancer with high growth and metastasis ability, we supposed that the overexpressed UHRF1 might be due to the low expression of tumor-suppressive microRNAs in these high metastasis cancers. However, the function of UHRF1 in ATC remains unknown and is worth researching deeply. In the present study, we showed that UHRF1 suppression was sufficient to repress the proliferation of ATC cells *in vitro* and *in vivo*. Previously, the expression of antiproliferative downstream effectors (p21 and Rb) of UHRF1 was inversely correlated with UHRF1 expression. UHRF1 knockdown induced cell cycle arrest at G1/S phase, which was consistent with the activation of the tumor suppressor genes [19, 40]. Furthermore, ATRA induced the cell growth inhibition and cell cycle arrest in G1 phase [41]. Additionally, the growth activity is low in well-differentiated thyroid cancer compared with undifferentiated thyroid cancer [42]. Thus, cell growth inhibition by UHRF1 suppression could be the results of the co-existence of cell cycle arrest and the differentiated status.

Numerous carcinogenesis models have been formulated over the past two decades to explore the cellular origin of thyroid cancer. One is a classic multistep carcinogenesis model. In this model, ATC is derived from well-differentiated thyroid cancer. The dedifferentiation process needs the accumulation of genetic mutations during the proliferation of mature thyroid cells [42]. Another one is fetal cell carcinogenesis model. This model emphasizes the pre-existence of tumor stem-like cells within the thyroid gland that can give rise to ATC. In our 3D culture model, ATC cells were differentiated when cells were knocked down of UHRF1. CD97 is recognized as a member of the adhesion family of G protein coupled receptors (GPCRs) and has been published to exert a critical role in promoting thyroid cancer progression in a mouse model [43]. Consistent with the above study, our results showed that CD97 was highly expressed in ATC cell lines and that UHRF1 inhibition reduced CD97 expression in undifferentiated cancer cells enhanced by PMA or ATRA treatment. Moreover, UHRF1 suppression could reduce the expression of stemness markers in ATC. Previously, microarray data analyses demonstrated that ATC exhibited upregulation of stem-like cells markers in comparison with PTC [44]. As UHRF1 was reported to be a transcription factor [40], and in our study, suppression of UHRF1 down-regulated CD97, Sox2, Oct4 and Nanog, thus we supposed that UHRF1 suppression could repress the dedifferentiation marker and stemness markers expression in a transcriptional level [45].

Tumor inflammatory reaction plays a crucial role in cancer formation and progression. Inflammation was reported to influence the growth and differentiation of thyroid [46]. Additionally, CD97 has a feature in signal transduction associated with the development or establishment of the inflammatory reaction [34]. In the present results, more immune cells were immersed in ATC than PTC, indicating that inflammatory microenvironment might contribute to the transformation of ATC. Cytokines are the key elements linking inflammation to cancer. For instance, chronic inflammation caused by IL-6 promoted the development colorectal cancer (CRC) [47] and the metastasis of lung cancer [48]. Autocrine IFN-γ was published to enhance the metastatic ability of breast cancer cells and contribute to the resistance to NK cells [49]. IL-1β secreted from microenvironment or the malignant cells enhanced the tumor angiogenesis and invasiveness [50, 51]. Recently, several studies suggested IL-8, TGF-α and TNF-α as interesting biomarkers of thyroid cancer [52–54]. Our results revealed that cytokines in ATC cell lines and tumor tissues, including IL-8, TGF-α and TNF-α, were down-regulated by suppression of UHRF1. Therefore, UHRF1 was essential in cytokine-related tumor inflammatory reaction. Moreover, numerous recent studies implicated that inflammation was stimulated by transcription factors (for example, NF-κB and AP-1), and that both NF-κB and AP-1 promoted the expression of cytokines (for example, IL-6 and IL-8) directly [55–57]. Thus, further studies are needed to explore whether UHRF1 induced inflammation is through the activation of inflammation-related transcription factors.

In this study, we found that inhibition of UHRF1 suppressed tumor growth both in a cell culture condition and in a xenograft mouse model. Importantly, UHRF1 inhibition could retard ATC progression by promoting cell differentiation and reducing inflammatory reaction, indicating that UHRF1 may be an attractive target for future ATC therapy.

## MATERIALS AND METHODS

### Tissue samples

14 paracarcinoma (7 PTC paracarcinoma and 7 ATC paracarcinoma), 14 PTC and 14 ATC clinical specimens used for IHC analysis and 3 paired paracarcinoma and PTC tissues used for Real-time RT-PCR were collected from thyroid cancer patients at Sun-Yat-sen University Cancer Center. Pertinent patient clinical reports were obtained with patient consent and the approval of the Institutional Clinical Ethics Review Board at Sun Yat-sen University Cancer Center.

### Cell lines and culture conditions

BCPAP, TPC-1, 8505c, Cal-62 and ARO cell lines were purchased from Guangzhou jenniobio Biotechnology Co.,Ltd. The PTC cell line BCPAP and ATC cell line ARO were maintained in Dulbecco's modified Eagle's medium (DMEM; Gibco) supplemented with 10% feta bovine serum (Hyclone). The PTC cell line TPC-1 and ATC cell lines 8505c and Cal-62 were maintained in RPMI 1640 (Gibco) supplemented with 10% fetal bovine serum (Hyclone). The cells were incubated at 37°C in a humidified chamber containing 5% CO_2_.

### Microarray analysis

Microarray data was obtained from NCBI Gene Expression Omnibus (GSE9115, http://www.ncbi.nlm.nih.gov/geo/query/acc.cgi?acc=gse9115, and GSE33630, http://www.ncbi.nlm.nih.gov/geo/query/acc.cgi?acc=gse33630) and the UHRF1 expression levels were analyzed with GEO2R (website: http://www.ncbi.nlm.nih.gov/geo/geo2r/?acc=GSE9115 and http://www.ncbi.nlm.nih.gov/geo/geo2r/?acc=GSE33630) and normalized to the mean value of respective normal controls.

### HE staining

HE staining was performed as previously reported [58].

### IHC assay and statistical analysis

Paraffin-embedded tissue specimens were sectioned, incubated in 65°C for 2 hours, deparaffinized in xylene and rehydrated. Antigenic retrieval was processed with PH 9.0 EDTA. The sections were then incubated in 3% H_2_O_2_ for 10 minutes, blocked in normal goat serum for 1 hour and incubated with an anti-UHRF1 antibody (1:100 dilution, Abcam) at 4 °C overnight. After incubation with the secondary antibody for 1 hour, specimens were incubated with H_2_O_2_-diaminobenzidine for 10 minutes. Sections were then counterstained with haematoxylin, dehydrated and mounted. Staining intensity and extent of UHRF1 expression were graded as follows: negative = score 0, bordering = score 1, weak = score 2, moderate = score 3, and strong = score 4. Extent of staining was grouped according to the percentage of high-staining cells in the cancer nest: negative = score 0, 1 to 25% = score 1, 26 to 50% = score 2, 51 to 75% = score 3 and 76 to 100% = score 4. UHRF1 scores were normalized to the mean score of paracarcinoma tissues.

### Plasmid construction and shRNA stable cell lines generation

Human UHRF1 was generated by PCR amplification and subcloned into the pcDNA6-myc-His B (pcDNA6B) vector. Knockdown of UHRF1 was performed with the specific shRNAs delivered by a lentiviral system purchased from Sigma-Aldrich Corp. according to the instruction manual. In brief, to generate the lentivirus containing UHRF1 shRNA, 293FT cells were co-transfected with PMD2.G and psPAX2 compatible packaging plasmids and pLKO.1 plasmid bearing the UHRF1 shRNA for 24 hours. Next, the cultured medium was gently refreshed and collected 48 hours later. To infect cells, cells were cultured with the lentivirus bearing specific shRNA in growth medium containing 8 μg/ml polybrene. Afterwards, cells were subcultured and selected with 4 μg/ml puromycin. The sequence of the shRNA constructs targeting the UHRF1 gene is: UHRF1 (NM_013282): shUHRF1-1-TRCN0000273313 (Insert Sequence: 5’-CCGGCGTCATTTACCAC GTGAAATA CTCGAGTATTTCACGTGGTAAATGACGTTTTTG-3’), shUHRF1-2- TRCN0000273315 (Insert Sequence: 5’-CCGG ATGT GGGATGAGACGGAATTG CTCGAGCAATTCCG TCTCATCCCACATTTTTTG-3’), shUHRF1-3-TRCN0000 273317 (Insert Sequence: 5’-CCGGGCGCTGGCT CTCAACTGCTTTCTCGAGA AAGCAGTTGAGAGCC AGCGCTTTTTG-3’). The MISSION Non-Target shRNA control vector SHC002 (Insert Sequence: 5’-CCGGCAACAAGATGAAGAGCAC CAACTCGAGTTGGTGCTCTTCATCTTGTTGTTTTT-3’) was used as a control.

### Real-time RT-PCR

Total RNA was extracted by using TRIzol reagent (Invitrogen), which was used to generate cDNA by using TranScript® All-in-One First-Strand cDNA Synthesis SuperMix (One-Step gDNA Removal) (TransGen Biotech). Real-time RT-PCR was performed using TransStart Tip Green qPCR SuperMix (2×) (TransGen Biotech) as recommended by the manufacturer. The primers used are listed in Supplementary Table S2. β-actin was used as the internal control.

### Western blot analysis

Cells were lysed by RIPA buffer and the Bradford dye method was used to determine the protein concentration. Equal amounts of cell protein were subjected to electrophoresis in SDS-PAGE gels and then transferred to PVDF membranes (Millipore) for antibody blotting. The following antibodies were used: UHRF1 (Abcam), Sox2 (epitomics), Oct4 (epitomics), Nanog (epitomics), β-actin (Cell Signaling Technology). Horseradish peroxidase-conjugated IgG (Pierce) was used as the secondary antibody.

### Plate colony formation assay

Approximately 500 cells were seeded into six-well plates and incubated for 7 days. Colonies were stained with crystal violet and counted.

### Cell viability assay

1×10^5^ cells per well were seeded in six-well plate and counted at the indicated time point by using a hemocytometer. Each cell line experiment was performed in triplicates.

### Nude mice xenograft assay

10 female nude mice (4 to 6 weeks old BALB/c athymic nude mice) were equally separated into two teams, one team was injected with control stable cancer cell line, while another team was injected with UHRF1 suppressed stable cancer cell line. Each mouse was injected with 1 ×10^6^ cells. The growth of tumors was measured every 3 days, and xenograft tumors were removed for final analysis. Volumes were estimated using the formula (a^2^×b)/2, where a is the shorter of the two dimensions and b is the longer one. The *p*-value is a comparison between the shC and shUHRF1 groups at the final time point. Care of experiment animal was approved by Institutional Animal Care and Use Committee of Cancer Center, Sun Yat-Sen University.

### 3D culture

Eight-chamberd RS glass slides (BD Falcon) were pre-coated with 50 μl/well of Matrigel (BD Biosciences) and solidified at 37°C for 30 minutes. Next, the cells were suspended in 2% Matrigel growth medium and plated at a density of 500 cells per well. The growth medium was refreshed every 3 days. The images were photographed after 10 days of culture by using an inverted microscope.

### CD97 expression analysis

To evaluate CD97 expression, 2 × 10^5^ cells (8505c shC, 8505c shUHRF1, Cal-62 shC and Cal-62 shUHRF1) per well were plated in six-well plate and were cultured with the indicated concentration of PMA or ATRA for the indicated times. The cells were then collected and incubated with anti-human CD97 (APC; eBioscience) at 4°C for 30 minutes in the dark and subjected to flow cytometric analysis.

### Statistical analysis

Each experiment was performed in triplicated for at least three times. Unless otherwise indicated, results were showed as mean ± SD of three independent experiments. Statistics were calculated by SPSS software (version 16.0) or GraphPad Prism 5 software by two-tailed Student's t-test. A *p*-value < 0.05 was considered statistically significant (*p<0.05, **p<0.01, ***p<0.001).

## SUPPLEMENTARY MATERIALS FIGURES AND TABLES


