# Supplementary material for: UHRF1 suppression promotes cell differentiation and reduces inflammatory reaction in anaplastic thyroid cancer
Source: Oncotarget. 2016 Jul 18;9(62):31945–57. doi: 10.18632/oncotarget.10674 (PMC6112835; doi:10.18632/oncotarget.10674)
Supplement: Supplementary file 1 [file oncotarget-09-31945-s001.pdf]

UHRF1 suppression promotes cell differentiation and reduces inflammatory reaction in anaplastic thyroid cancer

SUPPLEMENTARY FIGURES AND TABLES

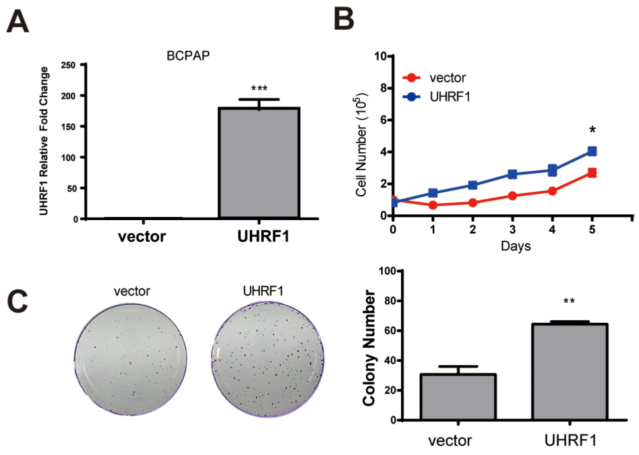

Supplementary Figure S1: Overexpression of UHRF1 in PTC cell line BCPAP promotes proliferation. A. PTC cell line BCPAP was overexpressed with UHRF1 or a vector control. Real-time RT-PCR was used to validate UHRF1 expression. (***p<0.001 by two-tailed Student's t test). **B.** Approximately 1×10^5 cells were seeded in each well of six-well plates. Cells were collected and counted by hemocytometer every 24 hours. All experiments were performed in triplicate. Error bars indicate mean ± SD (*p< 0.05, two-tailed Student's t test). **C.** The proliferation capacity of PTC cell line BCPAP was analyzed by colony formation assay. Representative pictures of the whole plates from triplicate experiments are shown. The mean ± SD of relative colony numbers was plotted (**p<0.01, values were calculated by two-tailed Student's test).

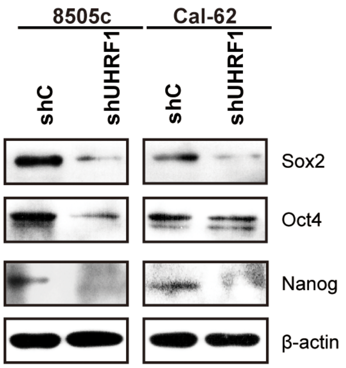

Supplementary Figure S2: UHRF1 knockdown reduces the protein expression levels of stemness markers in ATC cells. Western blot was used to validate the protein expression of Sox2, Oct4 and Nanog.

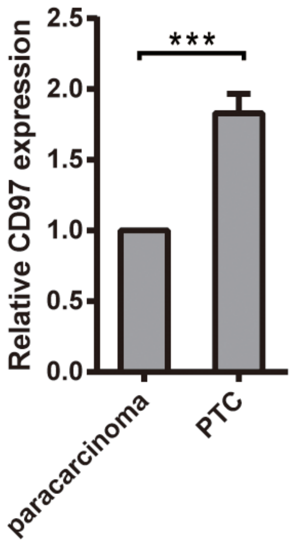

Supplementary Figure S3: CD97 is overexpressed in thyroid cancer compared with paracarcinoma tissue. CD97 mRNA expression of 3 paired paracarcinoma and PTC tissues was assayed by real-time RT-PCR. (****p*<0.01, values were calculated by two-tailed Student's test).

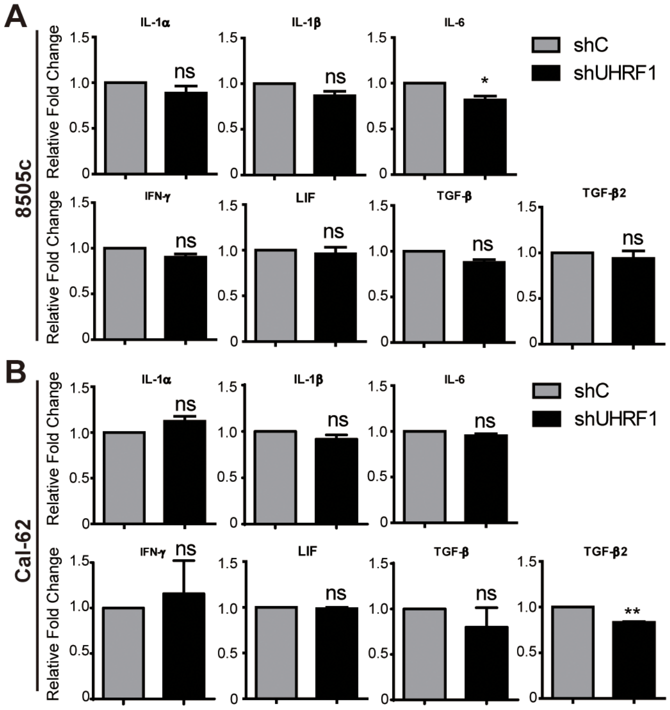

Supplementary Figure S4: Different cytokines expression in xenograft tumor cells after UHRF1 depletion. A. and **B.** Validation of different cytokines expression between shC group and shUHRF1 group in 8505c (A) or Cal-62 (B) xenograft tumor tissue by real-time RT-PCR. (**p*<0.05, ***p*<0.01 by two-tailed student's t test).

Supplementary Table S1: Expression pattern of UHRF1 in PTC and ATC tissues

Group	n	UHRF1	
		(-) 0-4	(+) 5-8
PTC	14	14	0
ATC	14	3	11
p		<0.0001	

UHRF1, Ubiquitin-like, containing PHD and RING finger domains, 1; PTC, papillary thyroid cancer; ATC, anaplastic thyroid cancer.
(-): negative-low, (+): positive-high. Pearson's χ^2-test.

Supplementary Table S2: Real-time RT-PCR primers

Primer	Sequence 5’-3’
IL-1α F	GCCCTCAATCAAAGTAT
IL-1α R	GTATCTCAGGCATCTCC
IL-1β F	ATGGCTTATTACAGTGGC
IL-1β R	GTAGTGGTGGTCGGAGA
IFN-γ F	ATTCAGATGTAGCGGATAA
IFN-γ R	TGTATTGCTTTGCGTTG
LIF F	GCCAACGGCACGGAGAA
LIF R	TTGCTGTGGAGGCTGAGGG
IL-6 F	TCCAGTTGCCTTCTCCC
IL-6 R	GCCTCTTTGCTGCTTTC
IL-8 F	ATGACTTCCAAGCTGGCCGTAGCT
IL-8 R	TCTCAGCCCTCTTCAAAAACTTCTC
TGF-α F	GCCCTGGCTGTCCTTAT
TGF-α R	AGCGGTTCTTCCCTTCA
TGF-β F	TTCCACCCACGCTACCA
TGF-β R	GCGGATTGACCACCCAGT
TGF-β2 F	GAGGAGCGACGAAGAGT
TGF-β2 R	TAGAAAGTGGGCGGGAT
TNF-α F	GAGTGACAAGCCTGTAGCC
TNF-α R	AAGAGGACCTGGGAGTAGAT
CD97 F	CAGCATCAGTGTGACAGCTC
CD97 R	CTATGAGGTGCCGGACAGGT
Sox2 F	AGCAACGGCAGCTACAGCATG
Sox2 R	GGACTTGACCACCGAACCCA
Oct4 F	GCCCGAAAGAGAAAGCGAACC
Oct4 R	CCCCCTGAGAAAGGAGACCCA
Nanog F	CCGAAGAATAGCAATGGTGTGAC
Nanog R	GGACTGGATGTTCTGGGTCTGGT
UHRF1 F	CCAGCAGAGCAGCCTCATC
UHRF1 R	TCCTTGAGTGACGCCAGGA
β-actin F	CATCCGCAAAGACCTGTACG
β-actin R	CCTGCTTGCTGATCCACATC
